# Phylodynamic analysis of the canine distemper virus *hemagglutinin* gene

**DOI:** 10.1186/s12917-015-0491-9

**Published:** 2015-07-25

**Authors:** Guan-Ming Ke, Chin-Hsiang Ho, Meng-Jung Chiang, Bintou Sanno-Duanda, Cheng-Shu Chung, Maw-Yeong Lin, Yong-Ying Shi, Ming-Hui Yang, Yu-Chang Tyan, Pao-Chi Liao, Pei-Yu Chu

**Affiliations:** Graduate Institute of Animal Vaccine Technology, College of Veterinary Medicine, National Pingtung University of Science and Technology, Neipu, Pingtung, Taiwan; Department of Veterinary Medicine, College of Veterinary Medicine, National Pingtung University of Science and Technology, Neipu, Pingtung, Taiwan; Country Year Limited Company, Taoyuan, Taiwan; Department of Medical Laboratory Science and Biotechnology, College of Health Sciences, Kaohsiung Medical University, Kaohsiung, Taiwan; Department of laboratory medicine, Edward Francis Small Teaching Hospital, Banjul, Gambia; Department of Medical Research, Kaohsiung Medical University Hospital, Kaohsiung, Taiwan; Translational Research Center, Kaohsiung Medical University Hospital, Kaohsiung, Taiwan; Department of Medical Imaging and Radiological Sciences, Kaohsiung Medical University, Kaohsiung, Taiwan; Department of Environmental and Occupational Health, National Cheng Kung University, Tainan, Taiwan; Department of Laboratory Medicine, Kaohsiung Medical University Hospital, Kaohsiung, Taiwan

**Keywords:** Canine distemper virus, *hemagglutinin* (H) gene, Spatiotemporal dynamics, Demographic dynamics, Taiwan

## Abstract

**Background:**

Canine distemper (CD) is one of the most contagious and lethal viral diseases in dogs. Despite the widespread use of vaccines, the prevalence of the CD virus (CDV) has increased at an alarming rate in recent years. In this phylodynamic study, we investigated the spatiotemporal modes of dispersal, viral demographic trends, and effectiveness of vaccines for CDV. A total of 188 full-length CDV *hemagglutinin* (*H*) gene sequences dataset were subjected to recombination analysis, including seven from modified live vaccine (MLV) strains and 12 from Taiwan specimens. After excluding the MLV strains and potential recombinant strains, alignments of 176 of 188 previous CDV strains were further used to analyze phylodynamic characteristics, and evidence of selection, and co-evolution.

**Results:**

The CDV genotype consisted of MLV-associated genotypes such as America-1 and Rockborn-like strains, which were characterized by long terminal branches and no distinct geographical patterns among lineages. In contrast, wild-type isolates clustered into lineages with a spatiotemporal structure and short terminal branches. Co-circulation and extensive diversification were simultaneously observed. The sequence variation signature was shaped by both geographic diversity and host tropism. Codon 506 was identified as a multi-epistatic interacting in the H protein.

**Conclusions:**

The topological signature revealed in this study suggests different epidemic scenarios. For example, a ladder-like backbone is a hallmark of directional selection, and cladogenesis at long terminal branches indicates the emergence of a surviving lineage. The stable effective viral population of CDV indicate the effectiveness of vaccines currently used to control the virus.

**Electronic supplementary material:**

The online version of this article (doi:10.1186/s12917-015-0491-9) contains supplementary material, which is available to authorized users.

## Background

Canine distemper (CD) is a highly contagious viral disease that affects many wild and domestic carnivores, resulting in high morbidity and mortality in immune-naïve hosts [1]. The mortality rate of the CD is second only to that of rabies among dogs [2]. Transmission occurs through contact or inhalation of contaminated fluids, although fleas have recently been reported as a vector for transmission [3]. The manifestation of CD varies from mild to severe symptoms depending on host immune status [2]. The incidence of CD in dogs has been drastically reduced by the use of modified live vaccine (MLV) strains [4], which have been highly effective for protecting dogs against CD in Taiwan. However, episodes of CD in vaccinated puppies were reported between 2005 and 2007 [5, 6], and prevalence among stray dogs was reported to be 18.2 % in 2006 and 3.6 % in 2011 [7]. Although several explanations have been proposed, the exact cause of vaccination failure remains unknown. Since MLV strains have become broadly used, their interaction with wild-type strains is an important issue that requires further exploration.

As a member of the *Morbillivirus* genus of the *Paramyxoviridae* family, CD virus (CDV), the causative agent of CD, shares many viral and infectious properties with the measles virus (MeV) [1]. Like all other morbilliviruses, CDV is an enveloped non-segmented single-stranded RNA virus. It contains a negative sense RNA genome that encodes six proteins. Of these, the H protein is a constituent of envelope glycoprotein spikes on the virion and initiates host cell entry by attaching onto cellular receptors such as signaling lymphocyte activation molecule (SLAM, CD150), or PVRL4 (Nectin4) [1, 8]. The H protein monomer is a type II transmembrane glycoprotein composed of a short N-terminal cytoplasmic tail followed by a transmembrane domain and a large C-terminal ectodomain. The ectodomain is structured as a stalk and a six-blade (B1–B6) β-propeller fold surrounding a central cavity [9]. Each blade module contains four-stranded anti-parallel β-sheets (S1–S4). Intriguingly, unlike other morbilliviruses, CDV is known to have a wide diversity of host species. The H protein has an essential role in cell tropism. Therefore, antigenic and sequence variation may affect the virulence, host range, and neutralization-epitopes of CDV. Substitutions at residues 530 and 549 of the H protein are reportedly crucial for influencing host membrane fusion and host switches [10–12]. Previous studies have revealed major differences between the wild-type and MLV strains with respect to the number and sequences of amino acids in the H protein [13]. For example, when a premature nonsense codon was introduced in the C terminus of the Onderstepoort (OP) strain, the H proteins of wild-type and OP strains encoded 607 and 604 amino acids, respectively [14]. Due to the unique structure of proline and the formation of disulfate bonds by cysteine, these residues have considerable impacts on the protein structures. Moreover, since post-translational glycosylation has important roles in the folding, processing, translocation, and expression of proteins on the cell surface, posttranslational glycosylation may be an essential factor in the efficacy of a vaccine developed to reduce the virulence of a virus [15]. The asparagine (N) residue in the consensus sequence N-X-S/T (where X can be any amino acid except proline) is a site of N-glycan chain addition [16]. Eight to nine N-linked sites are conserved in all *H* genes of field strains, whereas four (OP) or seven (Convac) sites are conserved in those of MLV strains. Of these, the N-linked sites at residues 309–411 are unique to wild-type strains [15]. Variations in the *H* gene may play important roles in antigenicity; accordingly, the H protein is often used to estimate genetic changes in CDV isolates, and its site-specific signatures should reveal the evolutionary trajectories of viral fitness.

Studies of the rapidly growing database of RNA viral gene sequences have revealed that, in addition to point mutations, recombination plays an important role in the evolution of non-segmented RNA viruses. The recombination frequency varies widely among RNA virus family [17]. The recombination frequency of negative-stranded RNA viruses is low, apparently as a direct result of their higher genome organization into ribonucleoprotein complexes [18]. However, recombination of CDV has been documented in the *H* genes [10, 19]. The *H* gene has the highest genetic variation in the viral genome and is therefore commonly used for molecular typing of CDV strains [20, 21]. CDV has geographically distinct lineages; strains that are within the same clade and share >95 % amino acid sequence similarity in the H protein are considered to be of the same genotype [22]. The clusters that were identified first were America-1 (vaccine strain) and −2, Asia-1 and −2, Europe-1/South America-1 (EU-1/SA-1), and Arctic clusters [23]. A further seven clusters have recently been identified: European wildlife (EW), South America (SA-2), Rockborn-like (RL, Vaccine D), Africa-1, Colombian (South America-3, SA-3) and another two Asian clusters, Asia-3 and −4 [15, 24–28].

Due to the widespread use of MLV vaccines in susceptible animals, and continuing reports of increasing host species range and emerging lineages [24–30], an improved understanding of the factors that affect lineage diversification is required, including the effects of viral demographics and evolutionary spatiotemporal transmission. It has been hypothesized that if viral phylogenies are shaped by epidemiological and evolutionary processes, then the population dynamics can reflect the condition of global transmission history and the effectiveness of prevention strategies. The *H* gene sequences of CDVs worldwide were analyzed, including those of 12 CDV isolates from puppies vaccinated during the 2005–2007 outbreaks in southern Taiwan. The Bayesian phylogenetic method was used to infer spatiotemporal aspects of the phylodynamic history of the *H* gene. The relationship between antigenic drift in the *H* gene and its epistatic interactions were also analyzed.

## Methods

### Ethics statement and virus isolation

All animal care and treatment procedures were conducted in accordance with legislation for the protection of animals. The protocol was approved by the Institutional Animal Care and Use Committee of Kaohsiung Medical University (Permit Number: 104016). CDV cases comprised 12 puppies treated at animal hospitals during 2005–2007 (Table 1). After the owners of the animals gave either their verbal or written consent to participate in this study, oculonasal discharge samples were collected from each puppy. Care was taken to minimize suffering by the animals in this and all other experimental procedures. The specimens were filtered through nitrocellulose paper with a pore size of 0.2 μm and inoculated in Vero cells [23].

**Table 1:** List of canine distemper virus (CDV) isolates analyzed in this study

CDV strains	Age of host dog (months)	Gender of host dog	Vaccination history	Clinical signs of host dog
K05-34-TW	9	F	Three shots	Unknown
K05-36-TW	12+	F	Boosted within 1 year	Rhinorrhea
K05-55-TW	7	F	Three shots	Respiratory distress, rhinorrhea, depression
K06-110-TW	2	M	One shot	Rhinorrhea
K06-117-TW	12+	M	Boosted within 1 year	Ocular discharge
K06-127-TW	12+	M	Boosted within 1 year	Respiratory distress, fever
PT05-13-TW	12+	F	Boosted within 1 year	Rhinorrhea, depression
PT05-15-TW	4	F	Three shots	Respiratory distress, anxiety, death
PT06-17-TW	12+	F	Boosted within 1 year	Respiratory distress, ocular discharge, anxiety, death
PT07-18-TW	12+	M	Boosted within 1 year	Respiratory distress, anxiety, death
CY05-S-TW	12+	F	Boosted within 1 year	Respiratory distress, depression
CY05-Y-TW	2	F	One shot	Rhinorrhea, ocular discharge

F: Female, M: Male. The CDV vaccine is typically included in a combined vaccine. Strains are designated by the abbreviation for the city where the strain was isolated and the outbreak number. For example, K36 indicates that the virus was isolated in Kaohsiung in the thirty-sixth outbreak

### Reverse transcription-polymerase chain reaction (RT-PCR) and sequencing of the full-length CDV *H* gene

RNA was extracted from 12 specimens using TRIzol reagent (Invitrogen, Carlsbad, CA, USA) according to the manufacturer’s instructions, and RT-PCR was performed using the Reverse-iT One-Step RNA PCR kit (ABgene, Epsom, UK) as previously described with primer pairs CD-HF (5′-CTC AGG TAC CCC AAC AAT GCT C-3′; residue position: 7063–7084) and CD-HR (5′-GCG CGG CCG CAA GCT TGA RAT GTG TAT CAT CAT AC-3′; 8905–8939) [31]. Primer pairs for amplifying and directly sequencing the CDV *H* gene were designed according to the published sequence of the OP strain (accession No. AF378705). The RNA extracted from the attenuated CDV vaccine served as the positive control. The expected 1877-nucleotide amplicons were sequenced with BigDye® Terminator v3.1 Cycle Sequencing Kits (Applied Biosystems, Foster City, CA, USA) on an ABI3730 DNA Analyzer (Applied Biosystems). All sequences were registered in GenBank under accession numbers EU296481 to EU296493.

### Alignment, detection of recombination and sequence variation

The CDV *H* gene sequence was aligned with all available *H* gene sequences in GenBank using Multiple Alignment with Fas Fourier Transform (MAFFT) with default settings [32]. After manually correcting and excluding sequences with ambiguously aligned codons, sequences without data for isolation date or location were also excluded. Further, sequences with the same animal host, isolation location, and isolation year were stratified by random sampling. A dataset of 1824-nt fragments (corresponding to residues 7079–8902 in the genome of the OP strain) from 188 strains were analyzed in terms of their recombination and phylogenetic relationships (Additional file 1). The dataset included the 12 Taiwanese strains, 169 worldwide strains reported in the past 40 years and obtained from GenBank, and seven MLV stains as reference. Since the MLVs were obtained by artificial egg- and cell-adapted processes, the isolation year and location for each prototype was used as a reference. Recombination Detection Program v3.44 (RDP) [33] and Simplot v3.5.1 [34] software were initially used to scan for recombination signals under default settings. The RDP method automatically explores and enumerates recombination signals in an alignment. This program used the RDP, GENECONV, and MaxChi methods for initial scanning of the recombination signals. The BootScan and SiScan methods were used to verify the scanning results. The cut-off for the p-value for multiple comparisons was set to 0.05. The phylogenetic relationships and genetic distances between potential recombinant strains and their reference strains were further demonstrated by a bootscan plot and simplot, respectively, by using the Simplot program. A potential recombinant strain was accepted only when it showed a significantly discordant clustering pattern in the phylogenetic analysis.

A new dataset with 176 *H* sequences was generated by removing the seven MLV strains and the potential recombinant strains identified from the previous dataset of 188 sequences. The coding sequence of the 176-sequences dataset was used for pairwise comparisons of p-distances in nucleotide and amino acid sequences in the MEGA6 program [35]. The relationship between the ratio of synonymous (silent, *dN*) and nonsynonymous (amino acid-altering, *dS*) mutations is the important indicator of selective pressure at the codon level, and indicates functional constraints on the maintenance of the encoded protein. When *dN*-*dS* > 0 (an overabundance of nonsynonymous substitutions; i.e., with *dN* > *dS* or *dN/dS* > 1) indicating a positive (or diversifying) selection. A neutral mutation or a negative (purifying) selection was indicated by *dN*-*dS* = 0 and *dN-dS* < 0, respectively. The Single Likelihood Ancestor Counting (SLAC) and the Mixed Effects Model of Evolution (MEME) were used to estimate site-specific selection. The SLAC is an effective method of rapidly identifying significant differences between *dN* and *dS* [36]. The MEME is a recommended method of detecting sites that have evolved under episodic selection pressure [37]. This method allows for the distribution of *dN*/*dS* to vary not only among sites but also among branches of a site. A p-value less than 0.05 was considered statistically significant when using both the SLAC and MEME methods. The Spidermonkey/Bayesian graphical model (BGM) method was used to detect sites with epistatic interaction [38], and a posterior probability (PP) < 0.5 was considered statistically significant. All programs used to detect selection, including SLAC, MEME and BGM, were implemented on the DataMonkey website [39].

### Phylodynamics analysis

Phylogenetic relationships were inferred by the neighbour-joining (NJ) and Bayesian Markov chain Monte Carlo (BMCMC) methods based on the full-length *H* gene sequence. The NJ trees were constructed using MEGA6. The best-fit model for both the 188- and 176-sequence dataset was the Tamura 3-parameter (T92) model [40] with a gamma distribution (five categories, [+G] parameter = 0.9494 and 0.9797 in the 188-sequence and 176-sequence datasets, respectively) using MEGA6. Tree reliability was estimated by bootstrap (BS) analysis of 1000 pseudoreplicate datasets.

The BEAST v1.8.2 [41] programs were used for BMCMC tree analysis, for which nodal support was estimated by calculating PP. The BEAST program can combine different substitutions, demographic scenarios, and clock models. Therefore, the tree analysis in this study included two model compositions: the Shapiro-Rambaut-Drummond-2006 (SRD06) model, which is a codon-based substitution model that allows base frequencies in the first or second codon to differ from those in the third codon and assumes a Hasegawa-Kishino-Yano model plus gamma-distributed rates (HKY + Γ) [42], and the Bayesian skyline plot (BSP) model, a non-parametric demographic model with either a relaxed uncorrelated lognormal distribution (ucld) clock model or a relaxed uncorrelated exponential distribution (uced) clock model. The use of a relaxed clock allows for each branch to have a different evolutionary rate. The co-estimates of the rate of growth, substitution rate, and mean time to the most recent common ancestor (T_MRCA_) were calculated for the MCMC runs using BEAST. According to the Akaike’s information criterion through Markov chain Monte Carlo simulation (AICM) [43] results obtained with the TRACER v1.6 program [44], the best model composition in both datasets was SRD06-uced-BSP. The statistical uncertainty in the data for each parameter estimate was reflected by the median value and the 95 % highest probability density (HPD). TRACER was also used to calculate effective sample size (ESS) based on the stationarity of the post-burn-in distributions and estimated parameters. An ESS value > 200 was interpreted as a convergence of a BMCMC sample on the posterior distribution. The maximum clade credibility (MCC) tree was produced and visualized using FigTree v.1.4.0 (http://tree.bio.ed.ac.uk/software/figtree/). A BEAST log file with rate indicators was generated by symmetric Bayesian stochastic search variable selection procedure on location reconstruction [45]. Non-zero expectancy rates, indicating a Bayesian factor (BF) > 3 were then estimated with the SPREAD application [46] and displayed using ArcGIS explorer (http://www.esri.com/software/arcgis/explorer-desktop). Discrete sampling locations (*K* = 20) were plotted as the center of the country from in which the strain had been isolated.

## Results

### Clinical and histological findings

A total of 12 isolates were collected from the rhinorrhea of puppies vaccinated during the 2005–2007 CD outbreaks in Taiwan. Mild-to-severe upper respiratory disease and neurological symptoms were the most common clinical manifestations and were noted in six of the 12 dogs. Common gross lesions included mild-to-severe conjunctivitis, rhinitis, and pneumonia. Three dogs which were dead on arrival showed demyelinative encephalitis in the white matter of the cerebellum, and further blood tests revealed lymphoid depletion.

### Detection of recombination of the *H* gene

**Fig. 1:**
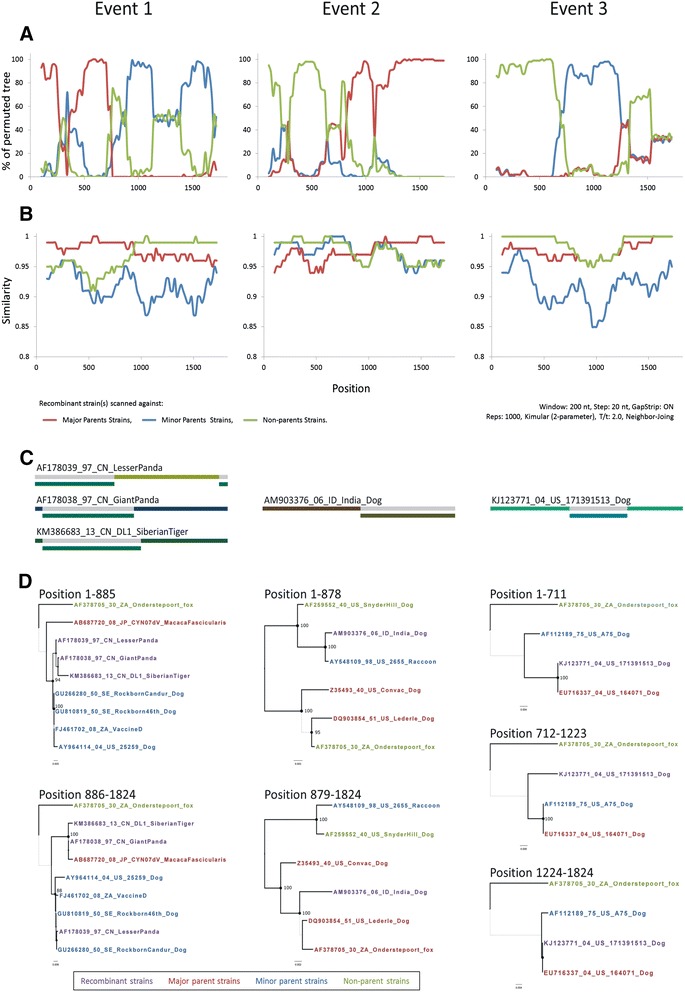
Recombination map of the canine distemper virus (CDV) *H* gene in strains of the 188-sequence data set. For each recombination event, the left side of the figure shows (**a**) the BOOTSCAN plot, and the second panel shows (**b**) the similarity plot. The y-axis shows (**a**) the percentage of permutation trees and (**b**) the pairwise identity of each pair in the sequence. The x-axis is the alignment position. The analysis was performed with a sliding window of 200 nt with a 20-nt step. The comparison was performed using 50 % consensus sequences with 1000 bootstrap replicates. Each curve in the figure compares the query and reference sequences. Both plots were generated with the Simplot program. Potential recombination breakpoints are identified where sudden alterations in bootstrap values or in similarity values occurred or where crossover occurred. Potential recombination breakpoints are identified where sudden alterations in bootstrap or similarity values occurred or where a crossover occurred. The third panel (**c**) shows a schematic diagram of potential recombinant regions and breakpoint locations identified by Recombination Detection Program v3.44 (RDP). Phylogenetic trees were compared by neighbor sequence fragment alignments flanking the breakpoint. (**d**). The potential recombinant strains, major parent strains, minor parent strains, and non-parent strains are indicated in red, blue, green, and purple, respectively

The 1824-nt CDV *H* gene sequences (encoding 607 amino acids) for the 188 examined strains were aligned to analyze sequence variation. Three recombination events with permutation rates > 70 % were identified by both RDP and Simplot programs (Fig. 1). Event 1 represents cross-species recombination. Three strains showed the same recombination pattern: AF178039_97_CN_LesserPanda, AF178038_97_CN_GiantPanda, and KM386683_13_CN_DL1_SiberianTiger. The major parent was AB687720_08_JP_CYN07dV_MacacaFascicularis (Asia-1), and the minor parents included all strains of RL genotype. However, the breakpoint in strain AF178039 differed from those of AF178038 and KM386683. Furthermore, the strain AF178039 was also identified as a minor parent of the latter two strains. Event 2 represents intra-genotypic recombination in the America-1 genotype. The AM903376_06_ID_India_Dog strain was identified as a recombination of the major parent strain AY548109_98_US_2655_Raccoon with the minor parent strains DQ903854_51_US_Lederle_Dog, AF378705_30_ZA_Onderstepoort_fox, and Z35493_40_US_Convac_Dog. The KJ123771_04_US_171391513_Dog strain was also identified as a potential recombinant of the major parent strain EU716337_04_US_164071_Dog and unknown minor parent strains. All of these recombination events showed highly discordant topologies between two neighboring segments flanking the breakpoint. After removing all of the potential recombinant strains and MLV sequences, the RDP program detected no recombination in this filtered 176-sequences dataset. Notably, the MLV strains in the America-1 and RL genotypes were apparently associated with recombination events 1 and 2, respectively.

### Phylodynamics analysis

**Fig. 2:**
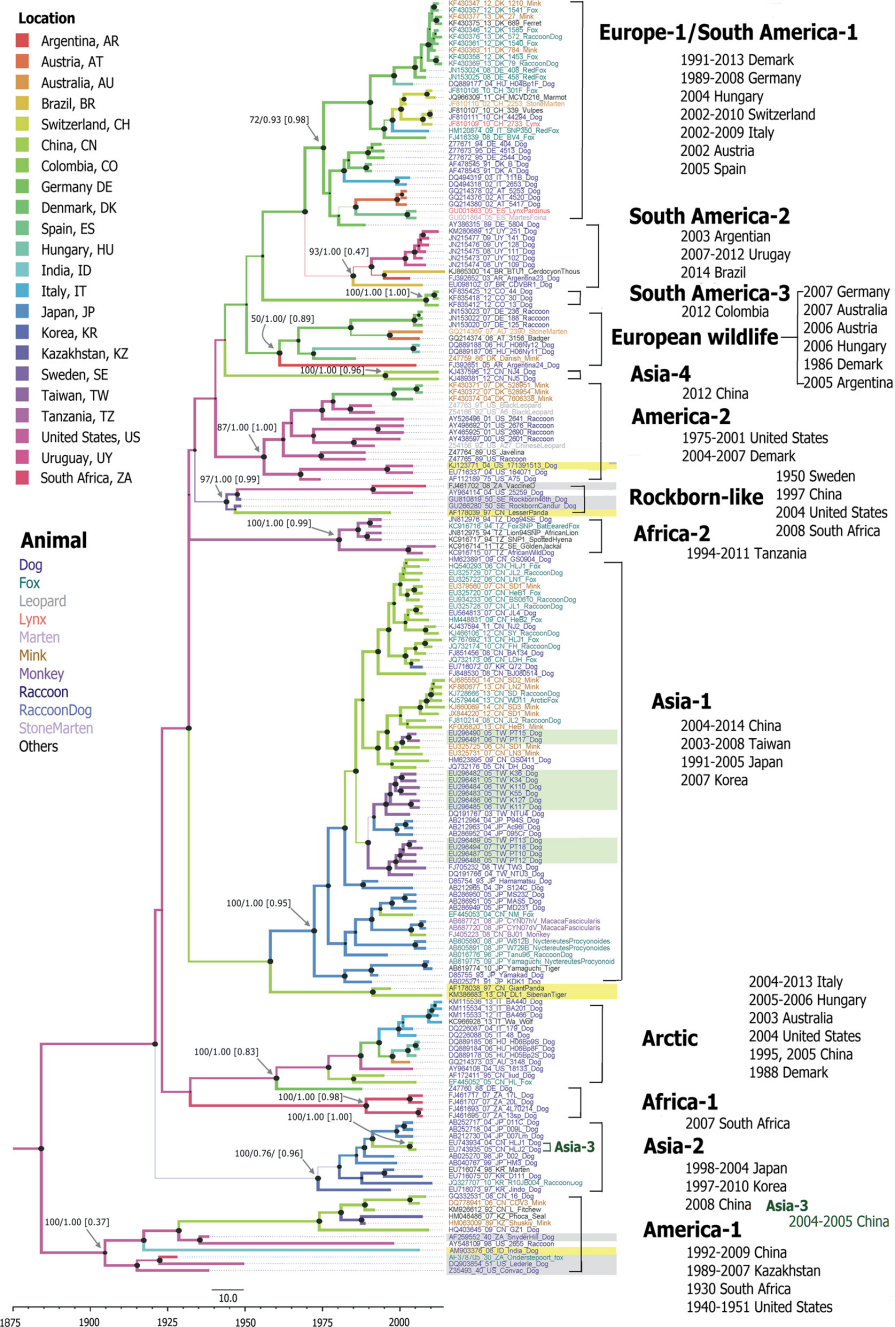
Maximum clade credibility tree based on 188 canine distemper virus *H* gene sequences. For each branch, the colour and thickness indicate the most probable location and most probable state, respectively. For key nodes, the size of the node cycle represents the posterior probability (PP), and support values are labeled above the nodes as either bootstrap (BS) or PP according to BS-NJ/PP-BEAST. Numbers in parentheses are the PP values of each location. The scale bar at the bottom shows the time in years. Strains are designated by accession number-year isolated (last two digits)-country abbreviation-strain name-host and are colored according to the host animal species. For each genotype, the geographic distribution and duration of isolation are also shown on the right. The modified live virus vaccine strains are shaded grey, potential recombinant strains are shaded yellow, and strains isolated in this study are shaded green. The tree was constructed using BEAST program

**Fig. 3:**
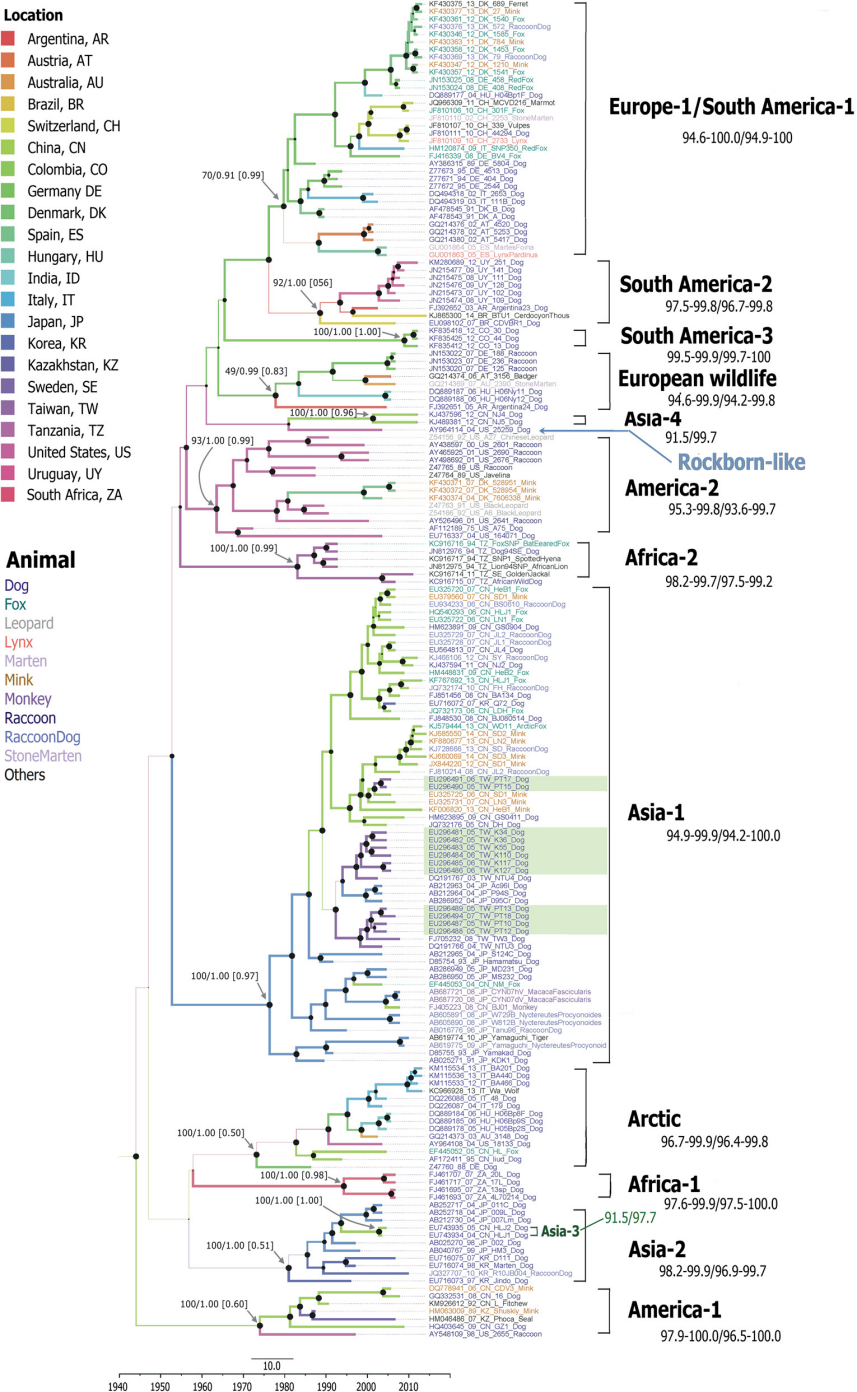
Maximum clad credibility tree based on 176 canine distemper virus *H* gene sequences. For each branch, the colour and thickness indicate the most probable location and probability of the state, respectively. For key nodes, the size of the node cycle represents the posterior probability (PP), and support values are labeled the nodes as either bootstrap (BS) or PP in BS-NJ/PP-BEAST. Numbers in parentheses are the PP values of each location. The scale bar on the bottom shows the time in years. The strain names are colored according to their host animal species. Genotypes and nucleotide/amino acid sequence similarities (%) appear on the right. Strains isolated in this study are shaded green

Phylogenetic relationships (among the 188- and 176-sequences dataset) were inferred by the NJ and BMCMC methods based on the full-length *H* gene sequences (Figs. 2 and 3). Phylogenetic trees constructed by the NJ and BMCMC methods showed similar genotypic groupings in both datasets. The CDVs grouped into a monophyletic cluster. All 14 distinct genotypes, which included America-1 and −2, Asia-1 to −4, Africa-1 and −2, Arctic, EU-1/SA-1, EW, RL, SA-2 and SA-3), and were depicted in this analysis (Figs. 2 and 3). In both datasets, almost all genotypes were supported by bootstrap (BS) and PP values of >70 and >0.9, respectively. The two exceptions were for EW in both datasets of the NJ trees and for America-1 in the 188- sequences dataset of the BMCMC tree. The analysis revealed that each genotype has continued to evolve, and isolates have been continuing identified and reported as recently as the past decade, including Italy strains in the Arctic genotype identified in 2012–2013 and Denmark strains in the EU-1/SA-1 genotype identified in 2011–2013. The Asia-3 genotype was identified as an extension of Asia-2 with high support values. Notably, the structure of the clusters tended to be more influenced by geography rather than by host range. All Taiwan strains, including the 12 isolates analyzed in this study, clustered into two subclusters in the Asia-1 genotype with high support values. This indicated that two clades of CDV strains had co-circulated in Taiwan: one major clade (grouped with Japan strains) and one minor clade (clustered with China strains).

Interestingly, the MCC tree topology showed a mixture of long and short terminal branches in each cluster (Figs. 2 and 3), whereas the NJ tree showed no difference in the lengths of terminal branches (Additional file 2). Two distinct topologies were observed: MLV genotypes (America-1 and RL) showed geographically unstructured long terminal branches, whereas others showed structured short terminal branches and the branches originated from the same outbreak in each cluster (Fig. 2). The topology for the non-MLV genotypes clearly revealed a ladder-like structure with geographic clusters. Geographically co-circulating lineages are very common. For example, Asia-1 and −2 strains have co-circulated in China and Japan; meanwhile, Arctic, EW, and other EU-1/SA-1 stains have co-circulated in several European countries. Both genotypes associated with MLV contained MLV strains and wild-type isolates. In the America-1 genotype, all wild-type isolates were clustered together with the Snyder Hill strain. Strains have been identified in United States (1998, raccoon), India (2006, dog), China (1992, fitches; 2006, mink; 2008 and 2009, dog) and Kazakhstan (1989, mink; 2007, seal). In the RL genotype, one strains was isolated in China (lesser panda, 1997) and one was isolated in the United States (2004, dog). The MLV-associated genotypes did not have geographically distinguishable topologies and were located in long terminal branches.

Although the relaxed clocked model was used to allow for a varying evolutionary rate in each branch, this analysis was based on the isolation dates and locations of the MLV prototype. A dataset (176-sequences) without sequences for MLVs and recombinant strains was also analyzed (Fig. 3). The branch length differed, the topology was similar from the full (188-sequences) (Figs. 2 and 3). However, the only one strain AY964114_04_US_25259_Dog of the RL genotype in the 176-sequences dataset was clustered with the Asia-4 genotype in the BMCMC tree (Fig. 3) and with the EW genotype in the NJ tree (Additional file 2). Support values were low in both trees. Analysis of the 176-sequences dataset revealed four dispersal routes with support values of BF > 3: (1) from the United States to Tanzania (BF = 3.277) in 1988 in the Africa-2 genotype; (2) from China to Taiwan (BF = 8.514) in 1998 in the Asia-1 genotype; (3) from Germany to Switzerland (BF = 64.160) in 2001 in the EU-1/SA-1 genotype, and (4) from Germany to Australia (BF = 14.866) in 2005 in the EW genotype. Additional file 3: Movie 1 shows an animation depicting the spatiotemporal spread of CDV.

### Population dynamics

**Fig. 4:**
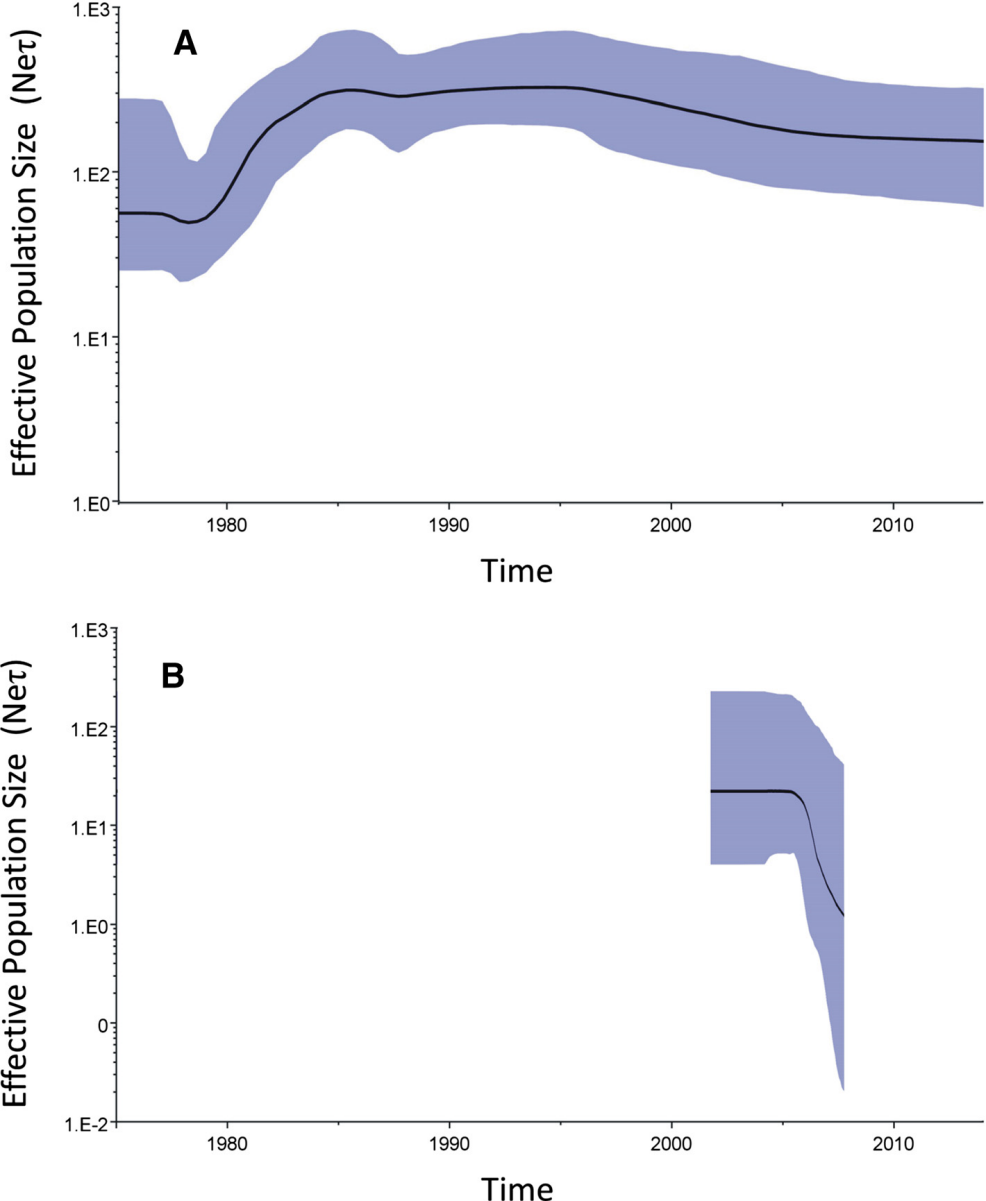
Bayesian skyline plot (BSP) of (**a**) 176-sequence dataset and (**b**) Taiwan strains isolated during 2003–2008 The x-axis is the time scale in years, and the y-axis is a logarithmic scale of *N*e*τ* (where *N*e is the effective population size and *τ* is the generation time). The thick solid line indicates the median estimates, and the shaded area indicates the 95 % HPD

Figure 4a shows the saddle-like BSP obtained for the 176-sequence dataset. For the first sampled strain (detected in 1978), the BSP revealed an effective population size (95 % HPD) of 56.1 (25.1–277.9). The BSP showed the occurrence of a population bottleneck in the early 1980s, although this may have been due to the limited number of sequences available for ancestor strains in GenBank. The population then peaked and plateaued (median population size range, 317.1–325.0) during 1985–1995, possibly due to advances in nucleotide sequencing technology and the consequent increase in the number of sequences reported worldwide. Since then, the viral population has consistently decreased (322.2–376.2) as vaccination programs and international cooperation have improved. After 2005, the population decrease was consistent (173.3–153.3), but the rate of decrease was relatively slow. The estimated T_MRCA_ was 1945 (1818–1966) and the evolutionary rate (95 % HPD) was 7.41 × 10^−4^ (6.00 × 10^−4^–8.80 × 10^−4^) substitutions per site per year (s/s/y). In the BSP for the Taiwan strains, the estimated initial effective median population size (95 % HPD) was estimated as 22.2 (4.0–227.4) in 2003. The population plateaued until 2005 and then consistently decreased from 22.2 to 1.2 during 2005–2008 (Fig. 4b).

### Detection of variation and selection of the *H* gene

**Fig. 5:**
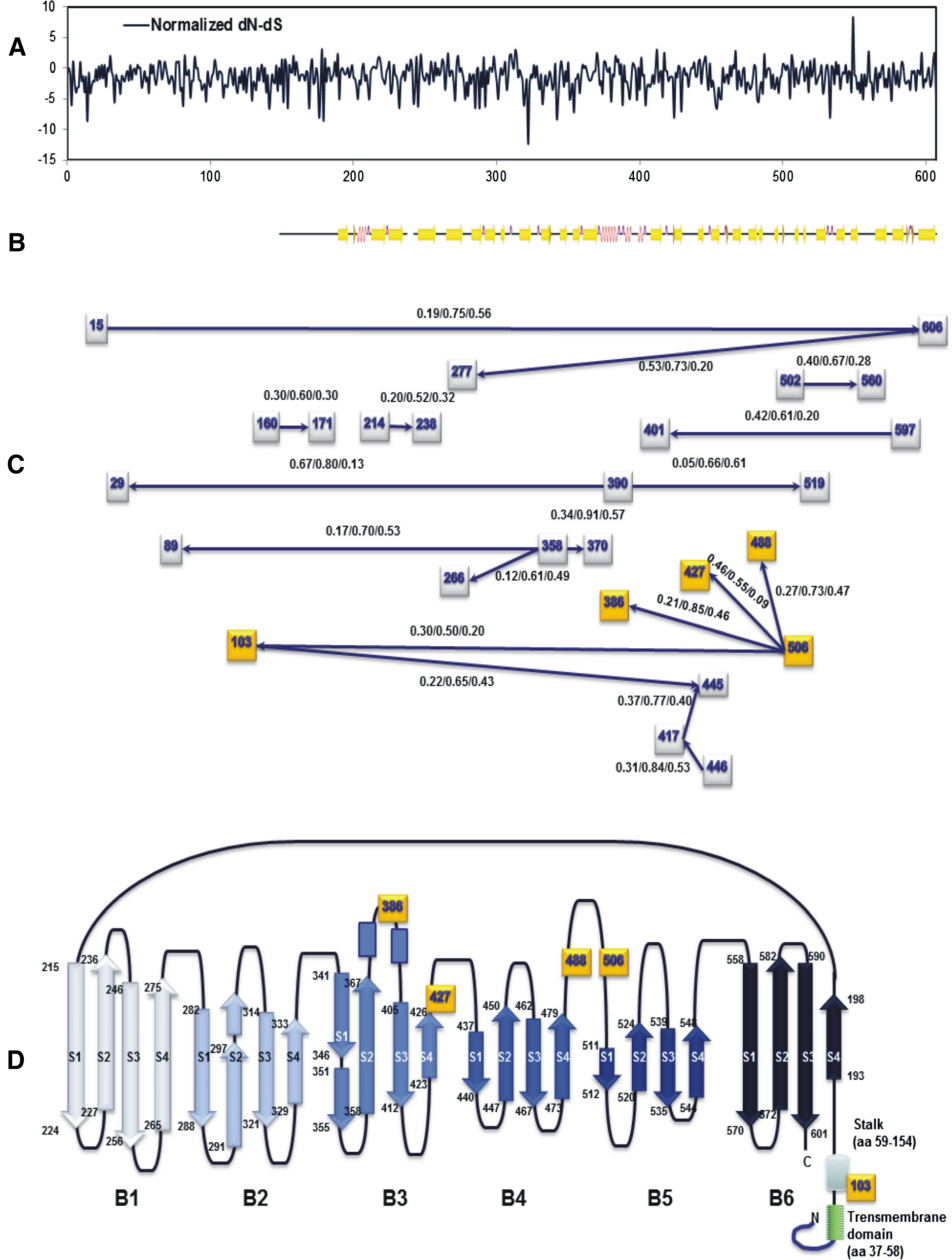
Site-specific variation among global canine distemper virus *H* sequences. **a** Detection of site-specific selection. Normalized *dN*-*dS* values were plotted for each codon site by using the SLAC method on the DataMonkey website. **b** Secondary structure guide for Measles virus (MeV) H protein (PDB ID codes 2ZP8). **c** Epistatic interactions among CDV H protein residues. Each square node represents the position of a residue in a CDV H amino acid sequence identified in at least one interaction. The analysis detected edges with marginal posterior probabilities (PPs) exceeding a default cutoff value of 0.5. Data are shown as PP(→)/PP(↔)/PP(←). The arrows indicate the directions of interactions. **d** The multiple epistatic sites detected in this study are labeled in the simplified secondary structure. Each amino acid residue and its coordinate structure are also denoted

The 1824-nt CDV *H* gene sequences (encoding 607 amino acids) for the 176 strains examined were aligned to analyze sequence variation and selection patterns. Pairwise comparisons showed that nucleotide and amino acid similarities for CDV strains were 87.2–100 % and 82.5–100 %, respectively. The amino acid sequence similarity was lower than 95 % between the EU-2, America-2, EU-1/SA-1, and Asia-1 genotypes (Fig. 3). The MEME method revealed 18 sites with evidence of episodic diversifying selection (P < 0.05): sites 15, 82, 150, 171, 175, 225, 254, 266, 275, 291, 310, 321, 368, 369, 372, 412, 537, and 549 (Additional file 4). Of these, positions 150 and 310 were in the second nucleotide of the potential glycosylation site, and positions 275 and 412 were proline residues in the OP strain. Ten of the these sites were located near the beginning (225 in B1S2, 266 in B1S3, and 291 in B2S2’) or near the end (254 in B1S3, 275 in B1S4, 321 in B2S3, 368 in B3S2, 412 in B3S3, 537 in B5S3, and 549 in B5S4) of the β-strand. Comparisons of deduced H protein amino acid sequences (residues 1–607) in different strains revealed that mutation patterns in Cys, His, glycosylation sites, and others had stronger associations with clusters than with isolation locations or hosts (Additional file 4). The Taiwan strains did not show any unique substitution patterns. Further, mutational signatures did not differ between MLV and wild-type strains or among different host species. However, viral isolates from non-human primates were clustered in a separate sublineage and had substitutions patterns unique to S24F, E276V, Q392R, D435Y, and I542F. Thus, the association between mutational signature and host tropism requires further study. Site-specific selection was analyzed using the SLAC and MEME methods. The SLAC method revealed one positive selection site (position 549) and 94 negative selection sites (P < 0.05) (Fig. 5a). Figure 5b shows the results of the BMG analysis, revealing 18 pairs of epistatic interactions (PP > 0.5). Interestingly, multiple epistatic interaction was detected at position 506 (with positions 103, 386, 488, and 427), and the relationships among these five residues are labelled in the secondary structure (Fig. 5c).

## Discussion

For effective prevention of an infectious disease such as CDV, a clear understanding of the pathway of spread is needed so that the transmission route can be blocked. The concept of phylodynamics postulates that viral phylogenies are shaped by both epidemiological and evolutionary processes [47]. Therefore, elucidating viral phylogenetic patterns could provide guidelines for reconstructing evolutionary histories of a virus, including spatiotemporal transmission and population dynamics [48]. As is the case for all phylogeographic studies, this study was limited by under- or oversampling of a given time and region, especially regarding the relative scarcity of ancestral strains (strains isolated before the 1980s). Furthermore, reliable data for specific isolation locations and dates were unavailable for many of the earlier strains. The locations for these strains were therefore plotted at the center of the country of isolation, and dates were presented in ranges of years. Therefore, conclusions can only be drawn according to the available sequence data. Notably, the absence of sequence data in GenBank does not rule out the possibility that these viruses existed at other times or locations. The situation is expected to improve as comprehensive global data accumulate in GenBank (e.g., data for Chinese strains recently submitted to GenBank include detailed information such as isolation date, isolation location, and host).

The AF178038 strain has been identified as a mosaic *H* gene resulting from a recombination event between the EW (EF445053) and Asia-1 (AF178039) genotypes [19]. This study revealed that the KM386683, AF178039, and AF178038 strains arose from recombination between the genotypes RL and Asia-1 (Fig. 1a). However, false-positive recombination signals can be generated artificially whereas false-negative recombinations might have occurred in unsampled parent strains [49]. It is worth noting that MLV strains in the America-1 and RL genotypes were apparently associated with recombinant events 1 and 2, respectively (Figs 1a and b). The KJ123771 strain appears to be a potential recombinant strain between the major parent EU716337 and unknown minor parents, and was also detected as a recombinant of the OP strain in the BootScan (Fig. 1c). In particular, event 1 was repeatedly detected in isolates, indicating that the potential interaction between MLV and wild-type strains should be under close observation. These recombination events can potentially affect viral host interactions, e.g., by extending the host range, increasing virulence, and promoting emergence of immune-escape lineages [29]. Therefore, these recombinants will require further study and continuous monitoring in the future.

This study and previous studies [50] of phylogenetic analyses of paramyxoviruses such as MeV or CDV have revealed several coexisting lineages or genotypes at any given time (Figs. 2 and 3). Outbreaks of these viruses occur in acute seasonal epidemic cycles, typically arising from repeated transmission in susceptible host populations. Infection or immunization results in strong lifelong immunity that is equally potent against all strains [51]. Researchers have postulated that survival of the remaining coexisting lineages is mainly driven by nonselective epidemiological processes [50]. However, a vaccination challenge revealed that by altering neutralizing epitopes, the MeV H protein may confer a selective advantage to surviving lineages [52]. Thus, both selective and nonselective processes can play important roles in the co-circulation of multiple lineages. Accordingly, the BMCMC trees in this study revealed a broad ladder-like backbone, even in the America-1 genotype. In contrast with MeV, in which humans are the sole natural host, a broad range of host species are susceptible to CDV infection, which complicates the selection pressure for this virus. This study further reveals that CDV has evolved into two topological classes: MLV-associated genotypes, such as America-1 and RL genotypes, in which lineages are tend to not geographically distinct; and wild-type isolates that cluster into geographically and temporally distinct lineages. Although the number of sequences for MLV-associated genotypes in GenBank is still too small to draw definitive conclusions. Since attenuated vaccines can maintain the strain in a stable condition, the long terminal branch may indicate that the genotype of this accidental strain maintained its genotype in its original form before MLV-associated strains evolved into a surviving lineage.

Previous studies have depicted genetic lineages of CDV, i.e., lineages with amino acid sequence similarity higher than 4 % [23]. The results of pairwise comparisons between CDV *H* sequence in this and previous studies showed comparable amino acid and nucleotide sequence similarity levels (>93 %) [53]. The estimated mean substitution for the *H* gene in CDV was 7.41 × 10^−4^ s/s/y, which indicated increased stability. In contrast, the mean substitution rate reported in Pomeroy et a. [54] was 11.65 × 10^−4^ s/s/y, possible due to the shorter duration of sampling (1982–2001) in their study. In some species, substitution rates remain constant because of the error-prone nature of RNA-dependent RNA polymerase. High mutation rates tend to decrease the stability of a protein. In terms of survival, however, the sequences tend to be conserved once reaching the fittest condition. That is, the substitution rate might be obscured by back mutations. The CDV *H* gene sequences from this study were compared with sequences and MLV strains isolated worldwide over the past 70 years and stored in GenBank. Of the nine potential Asp-linked glycosylation sites in the *H* gene [14, 15, 55, 56], all but two (amino acids 309–311 and 584–586) were present in old CDV vaccine strains (Additional file 4). A similar observation has been made for the *H* gene of MeV wild-type isolates as compared to the Moraten vaccine strain [57]. The glycosylation sites at amino acids 309–311 and 584–586 were present in wild-type isolates AB286953 and AY548109. Two old CDV wild-type isolates, AB472691 and FJ848531, had premature stop codons, suggesting that the amino acid number and absences of H protein glycosylation sites are unrelated to changes in virulence. While amino acid changes were scattered throughout the CDV H protein (Fig. 5a), they were mostly concentrated in regions surrounding and between glycosylation sites located at positions 309–311, 391–393, 422–424, and 456–458 [58]. This suggests that antigenic and selective pressures are higher at these sites. Although substitution at residues 530 and 549 of the H protein has a crucial role in host switching [10], the observed mutational trends indicate that a geographic signature cannot be ruled out. Epistatic interactions can also constrain evolution, with some mutations tolerated only after critical compensatory mutations. Although none of the interaction pairs found in this study (Fig. 5b and c) were located at the key binding sites of cell-surface receptors SLAM, such as positions 525, 526, 527, 528, 529, or 552, all of these sites have been reported as highly conserved residues [36, 59]. However, it is thought that the stalk region plays an important role in triggering the activation of fusion protein (F) during cell entry [60]. In addition, the deduced CDV and MeV H amino acid sequences show an identity of 36 % [61], the numbering for the H protein CDV residue is dephased by 4 to correspond with the numbering for the MeV H protein (e.g., CDV residue 506 corresponds to MeV residue 510). The multiepistasis site 506, identified in this study, corresponds to the site located near the B4-B5 hydrophobic groove in the H protein of MeV vaccine strains. This is a wide functional overlap regaion for the interaction with cellular receptor CD46 and PVRL4 [62]. This site is not only shows an epistatic interaction with 386, 423 (B3) and 488 (B4) but also interacts with site103 on the stalk region of the ectodomain.

## Conclusions

The spatiotemporal dynamics of CDV strains have been reconstructed by analysis of worldwide *H* gene sequences deposited in GenBank, and two types of CDV topologies were identified: the MLV-associated genotypes, i.e., America-1 and RL groups, were spatiotemporally unstructured with long terminal branches, and wild-type isolates were geographically structured with short terminal branches. Although an extension of the CDV host range was observed, the viral population and mutation rate has recently decreased considerably, possibly due to continuously improvement in international cooperation and an effective vaccination program. The MLV strains in both the America-1 and RL genotypes were confirmed as recombinational parents, and a small cluster at the branch terminal was found in the America-1 genotype. Therefore, the trend of an evolving surviving lineage of MLV-like strains should be under close surveillance. The results of this study are generally in agreement with those previous studies in that residues 530 and 549 have been subject to positive selection, although previous studies have reported interactions of sites with SLAM of host receptor. However, the mutational signature may be not only associated with host tropism but also with distinct geographic lineages. The key absorption residues in the H protein may be conserved to retain their vital function for host cell entry; therefore, a multi-epistasis site might indicate a new target for vaccine or drug development.

### Availability of supporting data

All the the supporting data are included as additional files.

## Additional files

Additional file 1:
**List of sampled canine distemper viruses (CDVs).** Live vaccine strains are highlighted in grey. Detected recombinant strains are highlighted in yellow. Strains analyzed in this study are shadowed in green. (XLS 113 kb) Additional file 2:
**Phylogenetic trees for canine distemper virus hemagglutinin (H) gene.** The neighbor-joining tree was conducted by (A) a 188-sequences dataset and (B) a 176-sequences dataset (nucleotides 7079–8902). Support values for main branches are given as percentages of bootstrap values (%) of bootstrap values in 1000 pseudoreplicates. The modified live virus vaccine strains are shaded grey, potential recombinant strains are shaded yellow, and strains in this study are shaded green. The tree was constructed with the Mega v6 program. (TIFF 9237 kb) Additional file 3:
**Movie-1.** Global spread of canine distemper virus. The lines between locations indicate the transmission route. Only the most plausible routes (BF > 3) are shown. Pushpins indicate the sampling locations, and the cycle size indicates the number of lineages at the sample location during the given time duration. (MOV 3047 kb) Additional file 4:
**Comparison of 607 deduced amino acid residues of CDV** ***H*** **sequences.** (A) Codon-specific selection sites are shown at the top; the x-axis shows the normalized dN-dS substitution values based on 176-sequence dataset obtained by SLAC method; significance selection sites (P > 0.05) identified by SLAC and MEME are labeled below. (B) Secondary structure guide for Measles virus H protein (PDB ID codes 2ZP8). Potential Asp-linked glycosylation sites (N-X-S/T) are indicated by asterisks (***) above residues 19–21, 149–151, 309–311, 391–393, 422–424, 456–458, 584–586, 587–589, and 603–605; Cys (○) and Pro (●) residues are also indicated. (C) Alignment amino acid sequences of H protein deduced from 188 sequences of CDV strains. Only the amino acids sequences that differed from those in the Onderstepoort strain are shown. Identical residues are indicated by dash. Vaccine-like strains are shadowed in grey, potential recombinant strains are shadowed in yellow and Sequences analyzed in this study are shadowed in green. Amino acids are color-coded as follows: black, nonpolar; purple, acidic; green, basic; blue, remaining polar amino acids. Sites at which Asp-linked glycosylation was lost are shaded cyan. (PDF 162 kb)

## Abbreviations

aa: amino acid
BF: Bayes factor
BS: Bootstrap
BSP: Bayesian skyline plot
CDS: Codon sequences
CDV: Canine distemper virus
H: Hemagglutinin
HPD: Highest probability density
MLV: Modified live vaccine
MCC: Maximum clade credibility
BMCMC: Bayesian Monte Carlo Markov Chain
nt: nucleotide
NJ: Neighbor-joining
OP: Onderstepoort strain
PP: Posterior probability
SLAM: Signaling lymphocyte activation molecules
TMRCA: Time to most recent common ancestor
ucld: uncorrelated lognormal distribution
uced: uncorrelated exponential distribution.
