# Supplementary figures and images for: Phylodynamic analysis of the canine distemper virus hemagglutinin gene
Source: BMC Vet Res. 2015 Jul 25;11:164. doi: 10.1186/s12917-015-0491-9 (PMC4513961; doi:10.1186/s12917-015-0491-9)

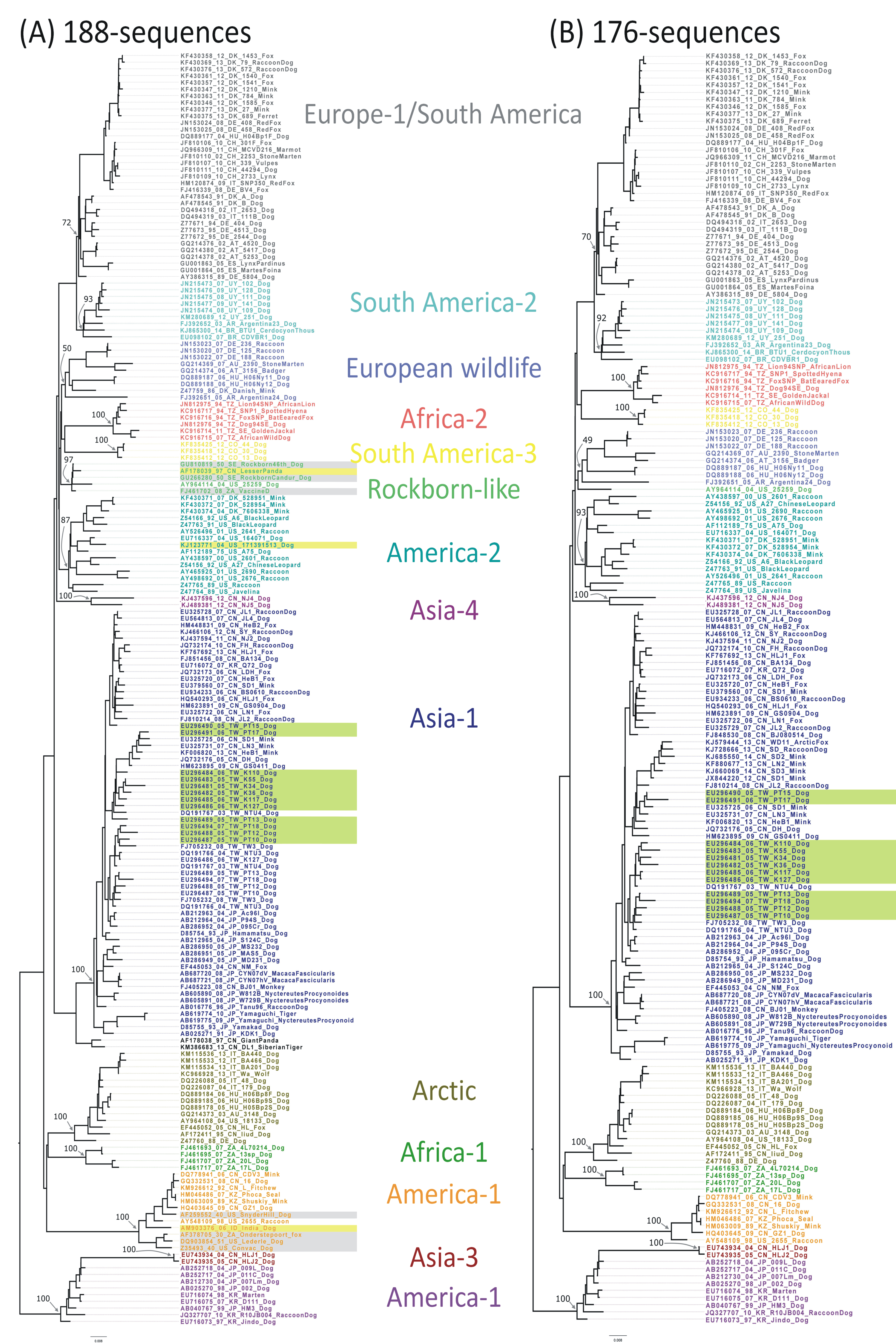

Supplement: Additional file 2: — Phylogenetic trees for canine distemper virus hemagglutinin (H) gene. The neighbor-joining tree was conducted by (A) a 188-sequences dataset and (B) a 176-sequences dataset (nucleotides 7079–8902). Support values for main branches are given as percentages of bootstrap values (%) of bootstrap values in 1000 pseudoreplicates. The modified live virus vaccine strains are shaded grey, potential recombinant strains are shaded yellow, and strains in this study are shaded green. The tree was constructed with the Mega v6 program. (TIFF 9237 kb) [file 12917_2015_491_MOESM2_ESM.tiff]

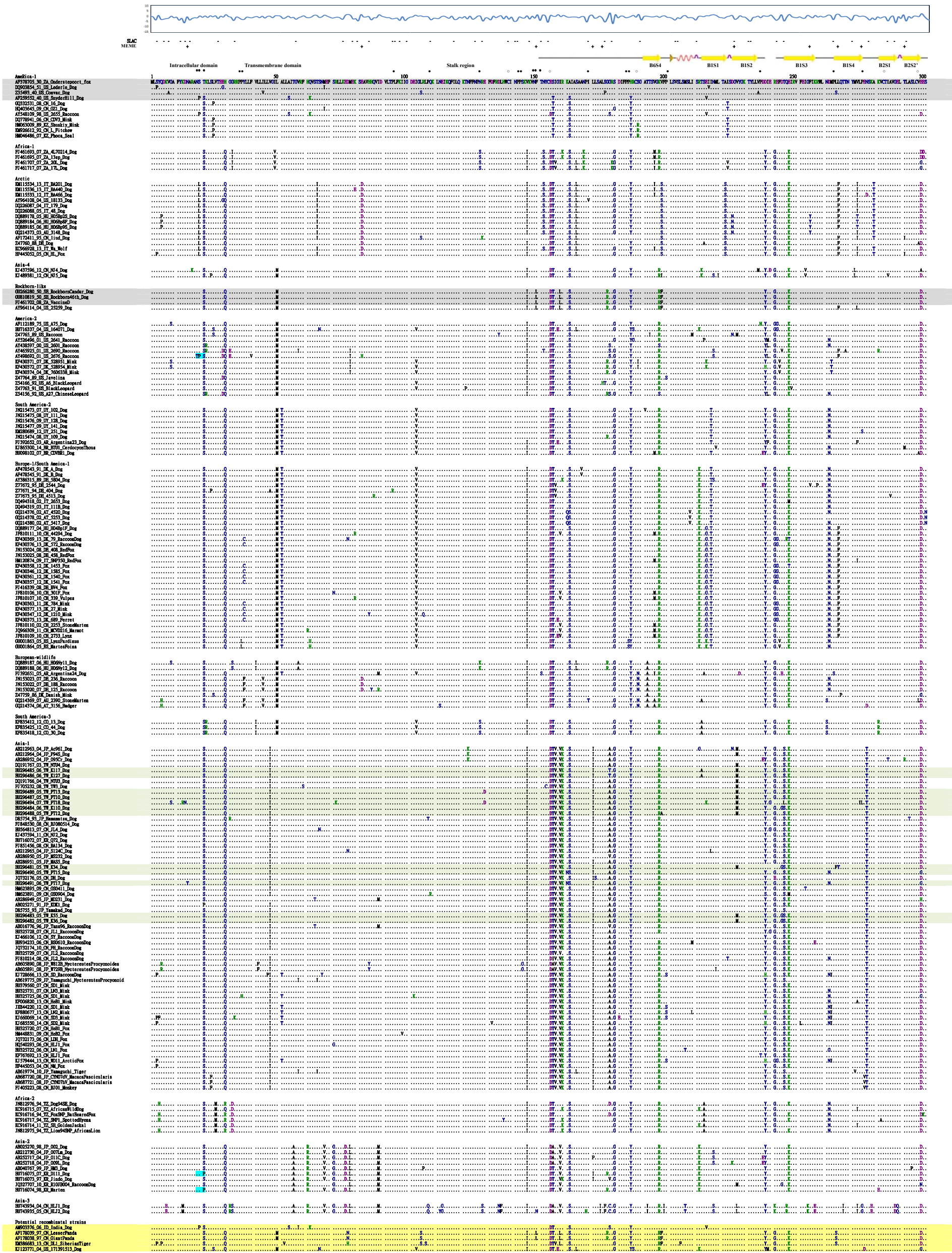

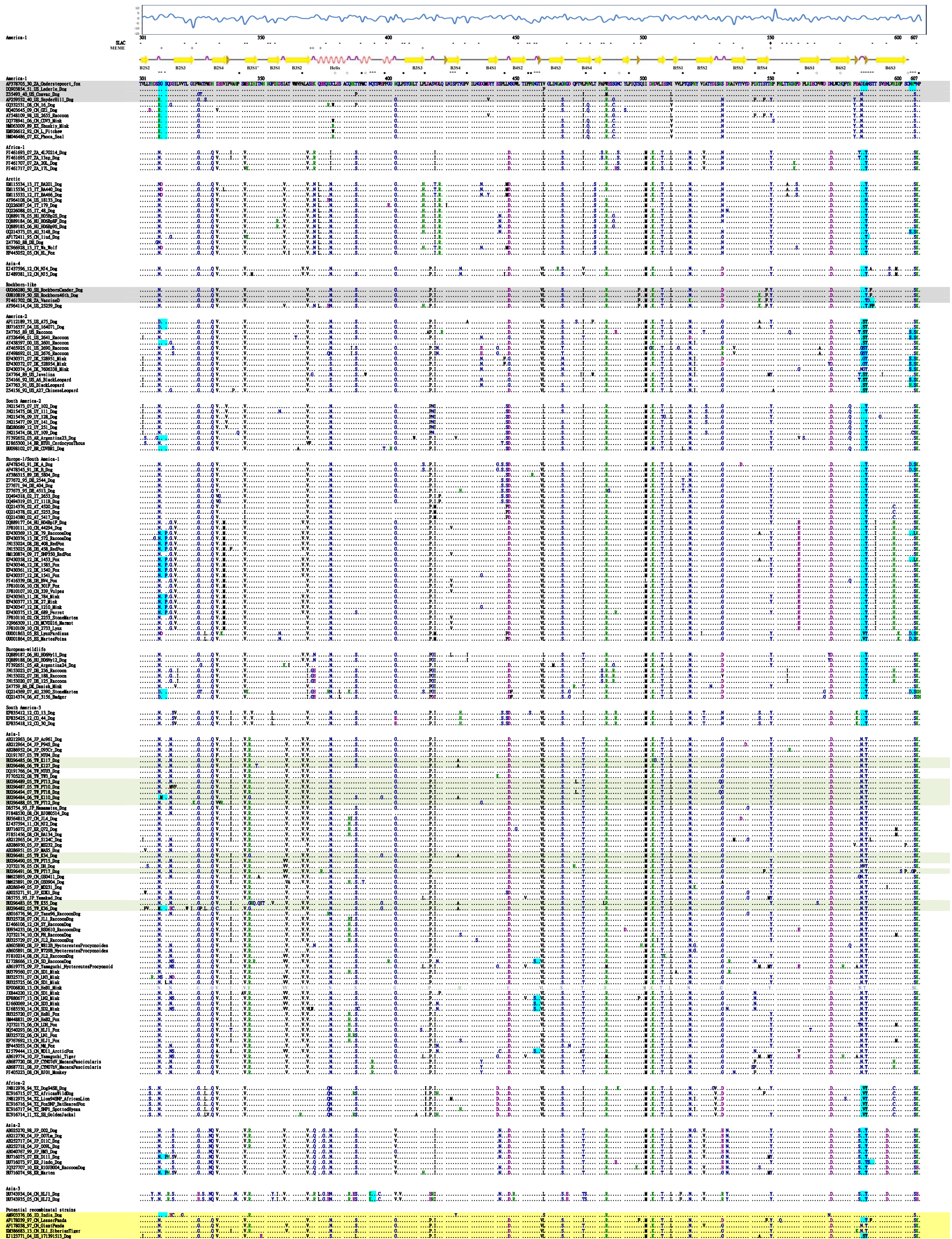

Supplement: Additional file 4: — Comparison of 607 deduced amino acid residues of CDV H sequences. (A) Codon-specific selection sites are shown at the top; the x-axis shows the normalized dN-dS substitution values based on 176-sequence dataset obtained by SLAC method; significance selection sites (P > 0.05) identified by SLAC and MEME are labeled below. (B) Secondary structure guide for Measles virus H protein (PDB ID codes 2ZP8). Potential Asp-linked glycosylation sites (N-X-S/T) are indicated by asterisks (***) above residues 19–21, 149–151, 309–311, 391–393, 422–424, 456–458, 584–586, 587–589, and 603–605; Cys (○) and Pro (●) residues are also indicated. (C) Alignment amino acid sequences of H protein deduced from 188 sequences of CDV strains. Only the amino acids sequences that differed from those in the Onderstepoort strain are shown. Identical residues are indicated by dash. Vaccine-like strains are shadowed in grey, potential recombinant strains are shadowed in yellow and Sequences analyzed in this study are shadowed in green. Amino acids are color-coded as follows: black, nonpolar; purple, acidic; green, basic; blue, remaining polar amino acids. Sites at which Asp-linked glycosylation was lost are shaded cyan. (PDF 162 kb) [file 12917_2015_491_MOESM4_ESM.pdf]
